# Integrating Lay Health Coaches Into Primary Care: Acceptability, Credibility, and Effectiveness From the Provider Perspective

**DOI:** 10.7759/cureus.25457

**Published:** 2022-05-29

**Authors:** Kira Reich, Susan W Butterworth, Mace Coday, James E Bailey

**Affiliations:** 1 Internal Medicine, University of Tennessee Health Science Center, Memphis, USA; 2 Center for Health System Improvement, University of Tennessee Health Science Center, Memphis, USA; 3 Preventive Medicine, University of Tennessee Health Science Center, Memphis, USA

**Keywords:** medical home, patient-centered care, psychosocial interventions, qualitative research, motivational interviewing, primary care, diabetes, lay health coaches, health coaches

## Abstract

The purpose of this mixed-methods, cross-sectional study was to assess the acceptability, effectiveness, and credibility of lay health coaches from the perspective of primary care personnel during coach integration into primary care teams through the Management of Diabetes in Everyday Life (MODEL) study. Surveys of 46 primary care clinic personnel were conducted in June 2017 and July 2017 to assess the acceptability, effectiveness, and credibility of lay health coaches in the clinics. Clinic personnel rated coach acceptability, impact, and credibility on a five-point Likert scale as 3.78, 3.76-4.04, and 3.71-3.95, respectively. Additionally, interviews revealed support for a team-based approach and recognition of the potential of coaches to enhance care. In the interviews clinic personnel also reported a lack of provider time to counsel patients as well as a need for improved provider-coach communication.

## Introduction

Effective management of diabetes mellitus places a significant daily demand upon patients. They must closely monitor their diet, glucose levels, medications, and comorbid conditions [1, 2]. These self-regulatory skills enable patients to minimize potential complications and improve overall quality of life [3, 4]. Despite the known risks of macrovascular (e.g., stroke, heart attack, and heart failure) and microvascular disease (e.g., blindness, renal failure, and amputation) associated with suboptimal management, following recommended self-care practices is particularly daunting for patients facing social and health inequities. As a result, many patients in medically underserved areas do not participate in or receive recommended care [5, 6].

Multiple studies have suggested that including non-clinician team members such as community health workers or health coaches can help patients with chronic conditions overcome barriers to diabetes self-care [7, 8]. While overall heterogeneous in methods, numerous studies have found that primary care-based lay health coaches can effectively assist diabetes patients in reducing average blood sugar (A1c) levels, improving vegetable and fruit consumption, and increasing physical activity. These studies suggest that benefits are similar whether primary care-based health coaches work with patients in person or via phone [9, 10]. Furthermore, most of the studies of health coaching in diabetes examined programs using lay health coaches without previous or extensive clinical training, suggesting that lay coaches can provide an effective and low-cost solution for patient lifestyle counseling.

While data from these clinical trials suggests that lay health coaches can be efficacious, it remains unclear whether the integration of these coaches into busy primary care teams is feasible or places additional burdens on other staff members. Little qualitative research is available documenting the experience of primary care team members with integrating health coaches into routine clinical practice. Further studies are needed to determine how adding lay health coaches to the practice teams affects providers, clinic personnel, and clinic workflow. Primary care clinics need qualitative implementation research results to assess the experience of primary care team members with coach integration into daily practice.

Our study employed the socio-ecological model to better understand the healthcare delivery system and patient-provider factors that may impact the introduction of diabetes health coaching in primary care settings [11]. This model utilizes a multilevel approach to analyze the personal, interpersonal relationship, community, and societal factors that can affect health and health behavior. Evidence-based health coaching approaches emphasize motivational interviewing, a patient-centered health counseling approach that fits well within the socio-ecological model. The current study uses the socio-ecological model to assess primary care providers and clinic personnel's experience and satisfaction with lay health coaches during their initial integration into primary care practices. The primary aim of the study is to assess the acceptability, credibility, and effectiveness of coaches from the perspectives of primary care team members including direct care providers and other clinic personnel. The information gained from this study will help to inform future approaches for integrating lay health coaches into patient-centered medical care.

Preliminary results from this study were presented as an oral presentation at the 2018 Society of General Internal Medicine Regional Meeting in New Orleans on February 23, 2018.

## Materials and methods

Design

This mixed-methods, cross-sectional study utilizes parallel and sequential self-administered surveys of all clinic personnel who agreed to participate and in-depth key informant interviews with providers in each of the four practices that participated in this study.

Setting

The study took place within the initial lay health coach integration and training phase of the Management of Diabetes in Everyday Life (MODEL) program [12]. MODEL is a Patient-Centered Outcomes Research Institute (PCORI)-funded pragmatic comparative effectiveness trial of health coaching and tailored motivational text messaging to improve diabetes self-care among African American adults with uncontrolled diabetes. As part of the MODEL program, lay health coaches were employed, trained, and certified jointly by participating clinics and University-employed MODEL study investigators and research staff. All coaches received intensive training in motivational interviewing (MI) and diabetes self-management in the targeted behavioral areas of healthy eating, physical activity, and diabetes medication adherence. The competency-based training focused on practicing evidence-based MI approaches for supporting self-efficacy of patients with uncontrolled diabetes to help them improve diabetes self-care behaviors.

Coaches were initially deployed in eight Mid-South primary care clinics; four clinics agreed to participate in the current study (three rural family medicine clinics and one urban endocrinology clinic providing primary care for patients with diabetes). Coaches were integrated into these clinics during and immediately following their training and certification. Continuing education sessions were held at each clinic on patient-centered care to orient all practice team members to the plans for training and deploying the health coaches, but they were not always well attended. No structured opportunities for provider observation of health coaches were provided, so levels of direct assessment of health coach efficacy by providers were variable. MODEL program health coaches began coaching participants in December 2016 and had been practicing for three to six months when the study began. The study was conducted from June through July 2017 (University of Tennessee Health Science Center IRB 16-04735-FB UM IAA).

Clinic personnel surveys

Survey information was gathered through an additional section of the second annual clinic personnel survey conducted within the larger MODEL study. Nine survey questions were included in both annual surveys and six additional questions about the health coaches were added the second year. Core survey questions were based on a review of the literature and expert consensus, then refined in iterative rounds to effectively capture key domains related to the integration of health coaches identified in the literature. Core survey questions assessed the level of agreement with positive statements regarding lay health coach acceptability, credibility, and impact. Responses were recorded on a five-point Likert scale ranging from 1 (strongly disagree) to 5 (strongly agree). The perspectives of providers were assessed through semi-structured interviews and then coded for common themes utilizing QSR International's NVivo qualitative data analysis software and a systematic framework approach [13].

The self-administered survey was distributed to clinic providers, direct care delivery staff, and other office staff in participating clinics. Quantitative data gathered from the surveys was analyzed using Statistical Analysis Software (SAS) Version 9.4 of the SAS System for PC, Copyright ©2017 SAS Institute Inc. SAS and all other SAS Institute Inc. product or service names are registered trademarks or trademarks of SAS Institute Inc., Cary, NC, USA. A copy of the statement of purpose and the questions relevant to this study are in Appendix A.

In-depth provider interviews

Qualitative in-depth interviews were designed and conducted using a phenomenological theoretical model [13]. The interviews took place in a private location and were recorded for audio-only. Each interviewer began with an explanation of the interview's purpose in the larger project, and assured participants their comments were both anonymous and would not lead to any negative repercussions for the health coaches.

The questions were developed with the help of experts on qualitative data analysis, health coaching, and clinic-based behavioral interventions using the socio-ecological model as a theoretical framework. The questions covered successes and challenges in managing lifestyle changes in patients with diabetes, overall impact, and experience with integration of health coaches, provider-health coach communication, barriers, and challenges in referring patients to coaches, adequacy of health coach training, relative value of lay health coaches compared with more highly trained clinical staff members, and value of the motivational interviewing approach. The full interview guide is in Appendix B.

Consistent with the phenomenological methodological approach, in-depth interviews were conducted in a free-flowing manner; the depth and coverage of follow-up questions varied depending on initial provider responses and provider time constraints. This approach allows researchers to define a phenomenon, which in this case is the experience of integrating health coaches into the primary care clinic, from the perspective of those who experienced it, and to define its meaning to them.

Interviews were recorded for audio-only and later transcribed for analysis. Audio files were stored in a password-protected Health Information Protection and Privacy Act compliant server and accessed only by the interviewing researcher. No other potentially personally identifiable information was collected (such as name, race, etc.) and recordings were deleted upon the completion of data analysis. After transcripts were checked for accuracy, they were then analyzed utilizing QSR International's NVivo qualitative data analysis software and a systematic framework approach.

Consistent with this approach, the audio from each interview was reviewed once prior to transcription, multiple times during transcription, and again afterwards. After becoming very familiar with the data, investigators identified and refined emerging themes. The data was then indexed according to the theme it fit best. Finally, the data was grouped by index and then from these groupings, summative flow charts were created to delineate the most salient themes [14]. The lead investigator, a medical student with a strong public health background and specific training in qualitative methods, served as the primary analyst of the qualitative data collected. Dr. Butterworth reviewed all of the qualitative data, worked simultaneously to identify themes, and verified the accuracy of indexing and the themes identified. Cases of disagreement were adjudicated through discussion and achieving consensus among all four investigators.

## Results

Clinic personnel survey

Out of approximately 70 total clinic personnel, a total of 46 potential participants were asked to complete a survey. Forty-five individuals completed the survey for a final response rate of 64.3%. The range of participants reflected the diversity of the participating practice teams and included nine physicians, two nurse practitioners, 14 nurses, nine medical assistants, eight front desk staff, three office managers, and one lab technician.

Clinic personnel's responses to survey questions are detailed in Table 1.

**Table 1:** Data Summary from Clinic Personnel Surveys Note: Each question corresponds to a specific domain and is worded as a positive statement. Responses were recorded on a 5-point Likert Scale with 1 corresponding to “strongly disagree” and 5 corresponding to “strongly agree.”

Data Summary from Clinic Personnel Surveys			
Question	Question Domain	Mean (N=45)	Standard Deviation
Lay health coaches have helped improve the quality of care my clinic provides to patients with diabetes.	Impact	3.78	1.16
My clinic has been able to integrate lay health coaches as part of the care team without difficulty.	Acceptability	3.78	1.26
Lay health coaches have been able to provide helpful counseling and support to our clinic’s patients.	Impact	4.04	1.09
Lay health coaches have been able to help our patients set and achieve their own diabetes self-care goals.	Credibility	3.95	1.07
Having lay health coaches assume some counseling responsibilities has reduced my personal workload.	Impact	3.76	1.19
Our diabetes patients who are working with a lay health coach have more productive visits with our clinic’s physicians and nurses.	Credibility	3.71	1.10

No significant differences in levels of agreement were found by clinic or type of provider (p > 0.05). Mean responses to questions assessing health coach impact on quality of care were 3.78 and 4.04 (range 1-5, with 4 indicating “Agree”). The mean response to the question assessing health coach impact on workload of other personnel was 3.76, and to the question assessing the acceptability of coaches 3.78. The mean responses to questions assessing clinic personnel perceptions of coach credibility were 3.95 and 3.71.

In-depth provider interviews

Provider Characteristics

Nine providers from the four clinics participating in the study agreed to participate in in-depth interviews. Most participating providers were primary care allopathic physicians (n=6), one was an endocrinologist providing primary care for patients with diabetes, and two were nurse practitioners. Most providers (n=8) practiced in suburban-rural practice locations and were female (n=6). Interviews ranged from 3.78 minutes to 17.72 minutes in duration with a mean duration of 10.3 minutes.

Supporting Lifestyle Changes in Patients With Diabetes

**Figure 1:**
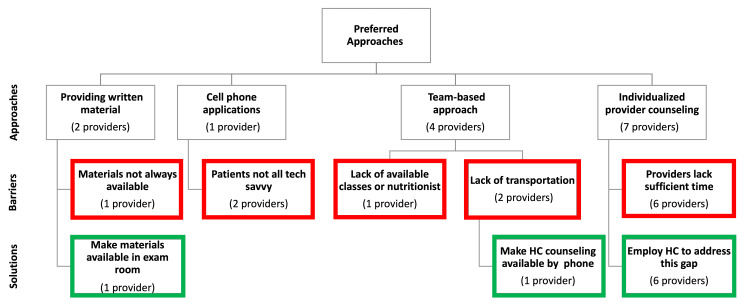
Preferred approaches for encouraging lifestyle choices for patients with diabetes including barriers and solutions

Providers reported finding a variety of techniques and/or tools useful for supporting lifestyle changes in patients with diabetes. Key themes identified through the interviews are displayed in Figure 1. Most providers (n=7) cited individualized provider counseling as their preferred technique for promoting lifestyle changes. However, almost all (n=6) noted that providers lacked sufficient time to do this effectively and health coaches were able to address this gap.

Overall Experience and Impact of Integrating Coaches Into Practice

**Figure 2:**
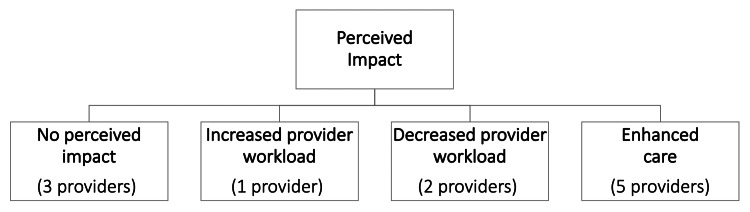
Provider perception of the impact of integrating health coaches into their practice

Key themes regarding early provider experience with MODEL program health coaches and their perceived impact are detailed in Figure 2.

Most providers (n=5) reported health coaches enhanced the care provided to diabetic patients. Two of the nine reported they did less counseling themselves and allowed health coaches to take over more of that responsibility. One provider reported that health coach integration resulted in an initial increase in workload because of the need to refer eligible patients to the health coach. However, the provider noted that these increased workload requirements resolved when the coach began proactively identifying eligible patients, encouraging provider referral, and offering services to eligible patients without referral. Three providers reported no perceived impact from the health coaches on patients but speculated insufficient communication with the health coaches after the initial patient referral to coach was partly to blame. So they felt unable to accurately comment on the impact of the health coaches.

Provider and Health Coach Communication

Provider-health coach communication problems were spontaneously reported in eight of nine provider interviews even though they were not asked about this topic. Two providers reported that they met regularly with the health coaches, typically in response to coach questions about individual patients. The remaining six reported no real communication with coaches following patient referral.

Barriers and Challenges in Referring Patients to Health Coaches

**Figure 3:**
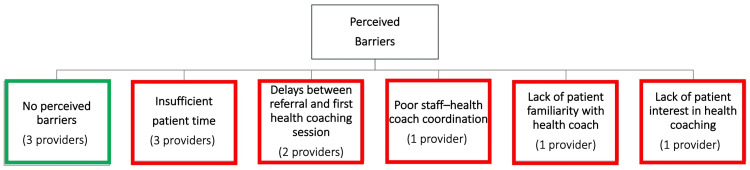
Perceived barriers to referring patients to health coaches

Although many providers initially reported no barriers or challenges to patient referral for health coaching, most providers did identify barriers in response to follow-up questions. Key themes related to referral barriers and challenges are detailed in Figure 3.

Some providers offered potential solutions to these barriers. For example, two suggested that health coaches take the initiative and identify patients likely to benefit from coaching without requiring provider referral. Another suggested that posters featuring the health coach’s face would increase engagement with patients.

Summary of Themes From Provider Interviews

As detailed in Figures 1-3, four main themes emerged from the analysis of in-depth provider interviews. They included support for a team-based approach and recognition of the potential of coaches to enhance care. Other themes were a lack of provider time to counsel patients and a need for improved provider-coach communication.

## Discussion

This exploratory mixed-methods study is among the first to document the general acceptability, credibility, and effectiveness of integrating lay health coaches into primary care practices from the perspective of primary care clinic personnel. Surveys of personnel in clinics in medically underserved areas indicated practitioners and staff generally have more positive than negative perceptions regarding lay health coach acceptability, credibility, and effectiveness. Primary care personnel, while not overwhelmingly enthusiastic, were generally open to integrating lay health coaches into their clinical teams and perceived long-term potential benefits. In-depth interviews of providers were consistent with survey findings and revealed four salient themes. They included support for a team-based approach and recognition of the potential of coaches to enhance care. Other themes were a lack of provider time to counsel patients and a need for improved provider-coach communication. Overall, these findings suggest that there is general support for integrating lay health coaches into primary care practice teams, but practical implementation approaches are needed to improve communication between health coaches and clinic personnel. Our findings indicate that improved implementation strategies for integrating health coaches that emphasize effective team communication are likely to result in improved acceptability, credibility, and effectiveness of health coaches from the provider perspective.

The current study’s generally positive results regarding lay health coach acceptability, credibility, and effectiveness are consistent with the existing literature regarding health coaches and community health workers. Specifically, we found that the findings from previous studies indicating the effectiveness of health coaches and other community health workers [7, 8] were affirmed from the perspectives of primary care team members in primary care practice settings where these non-clinician team members were being integrated. Providers and staff viewpoints agreed with these previous studies indicating that lay coaches can provide an effective and low-cost solution for patient lifestyle counseling to improve diabetes outcomes. Interestingly, our health care personnel survey results did not differ based upon the respondent type, suggesting that various personnel types are fairly uniform in their views regarding the value of integrating lay health coaches into the primary care team. Further investigation into variations in perceptions and attitudes toward lay health coaches among health care personnel types may prove useful for their future integration into established teams.

We chose to look at perceived acceptability, because provider and clinic personnel buy-in is an imperative component of sustainable primary care-based interventions. The first theme that arose from our interviews was significant support for a team-based approach to managing diabetic patients. This speaks to the overall acceptability of efforts to integrate lay health coaches into primary care practice. A team-based approach has also been identified as a prerequisite for patient-centered medical homes providing excellent primary care [7, 15]. A review of 30 innovative primary care practices, as nominated by experts in the field, found that 50% of these practices employed lay health workers who worked as coaches, care coordinators, or community health workers [15]. Our results indicating strong health care personnel support for a team-based approach employing lay health coaches are consistent with previous studies showing that effective teams decrease rates of burnout for clinic personnel and improve patient satisfaction [16, 17].

The current study’s findings that providers lack sufficient time to effectively counsel patients and they welcome assistance with counseling from lay health coaches are also consistent with previous research. Providers have cited time constraints as a major barrier to providing effective health counseling in numerous previous studies [18, 19].

We found a number of limitations in the study, however. The lack of complete anonymity in the clinic personnel survey could have resulted in positively biased responses, since participants may have felt uncomfortable critiquing their coworkers. Also, this mixed-methods study assessed clinic personnel perceptions only, and did not evaluate any specific quantitative measures of health coach effectiveness in improving patient care. Furthermore, only half the clinics enrolled in the parent study agreed to participate, and they may likely be clinics that more successfully integrated health coaches. Thus, the results of this exploratory study should be interpreted with caution. Even so, the study included numerous health care personnel from four diverse clinics in medically underserved areas and was consistent with previous studies, suggesting that the primary study findings regarding provider and staff attitudes towards lay health coaches are likely to be robust and generalizable. Finally, it is possible that poor provider-health coach communication resulting from inadequate coach training in the use of electronic medical record (EMR) electronic messaging systems may have negatively biased provider attitudes towards health coach integration. It is likely that better implementation of standard communication procedures would have improved provider attitudes regarding the benefits of a health coach.

The current study also found that providers consistently believed that lay health coaches enhanced overall patient care. We found little evidence that low perceived credibility and effectiveness of lay health coaches could potentially impede their utilization and integration into practice teams. Both the providers and other health care personnel we studied consistently recognized lay health coaches as credible and potentially effective providers of health counseling services. Given the growing body of evidence that lay health coaches can have positive effects on healthy behaviors, improved awareness of and exposure to health coaches is likely to continue to improve their perceived credibility and effectiveness [9, 10].

Our study also provides key guidance for implementation efforts to integrate lay health coaches in clinical practices, demonstrating that integration is supported by standardized workflow processes that emphasize effective provider-health coach communication in person and through the electronic health record (EHR). Optimized EHR utilization has been seen as a key feature of innovative primary care practices [15]. This study clearly shows that providers need effective channels, protocols, and methods for communication with lay health coaches, particularly through the EHR [20, 21]. Our results suggest that improved provider-coach communication is likely to increase provider confidence in coach credibility and effectiveness. In addition, this study suggests that health coach integration is facilitated through the adoption of standardized workflow processes whereby health coaches proactively identify eligible patients, encourage provider referral, and offer services directly to eligible patients without a referral.

## Conclusions

This study documents that primary care personnel generally perceive lay health coaches as an acceptable, credible, and effective intervention to improve care for vulnerable patients with diabetes. However, to maximize the value of this intervention, provider-health coach communication and coordination must be improved and standardized workflow processes are needed. Providers, while not principally opposed to direct in-person communication with health coaches, suggested that communication through the EMR is the most time-efficient for ensuring effective provider-health coach communication. Future studies should further evaluate provider perspectives on health coach integration into the primary care team in settings where standardized provider-health coach communication procedures are implemented systematically from the outset.

## Appendices

Appendix A: Clinic Personnel Survey - Health Coaching Related Questions Only

TITLE: Assessing Best Practice in Primary Care-based Delivery of Low Literacy Diabetes Education Materials to Vulnerable Patients in Medically Underserved Areas

PRINCIPAL INVESTIGATOR: James E. Bailey, MD, MPH

Professor of Medicine and Preventive Medicine

Coleman Building, Room D227

Memphis, TN 38163

Phone: 901-448-2475

CO-INVESTIGATORS: Drs. Helmut Steinberg, Betsy Tolley, Macy Coday, Susan Butterworth, Carolyn Graff, and Mary Lou Gutierrez; and Alex Galloway, Ian Michalak, Nick DiLoreto, Aubrey Flowers, Kira Reich, Stanley Dowell, and Zachary Pope

This survey is being conducted as part of a research study by the UTHSC Center for Health System Improvement. The purpose of this survey is to collect data about diabetes educational materials, use of lay health coaches, and improve diabetes care. This survey is designed to assess your clinic’s current use and distribution of diabetes educational materials, your preferences for improvements to diabetes educational materials, and your clinic's proficiency in caring for individuals with low health literacy. To obtain valid results, this survey should be completed by at least 50% of clinic personnel in each of the following categories: 1) Provider, 2) Nursing, and 3) Office staff.

The survey will take about 10 minutes to complete. Participation is voluntary and will not adversely affect your rights or your employment status. There will be no identifiable information about you shared as a part of this process. All information collected in the study will be kept confidential, and all recorded materials on a password-protected computer and in a locked file cabinet only accessible to key study personnel. If you have any questions or concerns, please contact Dr. Jim Bailey at 901-448-2475. Thank you for your participation.

**Table 2:** Clinic Personnel Survey on Health Coach Integration into Primary Care Practice Team

	Strongly Disagree	Disagree	Neutral	Agree	Strongly Agree
Lay health coaches have helped improve the quality of care my clinic provides to patients with diabetes.	☐	☐	☐	☐	☐
My clinic has been able to integrate lay health coaches as part of the care team without difficulty.	☐	☐	☐	☐	☐
Lay health coaches have been able to provide helpful counseling and support to our clinic’s patients.	☐	☐	☐	☐	☐
Lay health coaches have been able to help our patients set and achieve their own diabetes self-care goals.	☐	☐	☐	☐	☐
Having lay health coaches assume some counseling responsibilities has reduced my personal workload.	☐	☐	☐	☐	☐
Our diabetes patients who are working with a lay health coach have more productive visits with our clinic’s physicians and nurses.	☐	☐	☐	☐	☐

Appendix B: Provider In-Depth Interview Guide

TITLE: Clinic Personnel Perspectives on the Use of Lay Health Coaches to Improve Self Care Skills in Patients with Diabetes

PRINCIPAL INVESTIGATOR: James E. Bailey, MD, MPH

Professor of Medicine and Preventive Medicine

Coleman Building, Room D227

Memphis, TN 38163

Phone: 901-448-2475

CO-INVESTIGATORS: Drs. Helmut Steinberg, Betsy Tolley, Macy Coday, Susan Butterworth, Carolyn Graff, and Mary Lou Gutierrez; and Alex Galloway, Ian Michalak, Nick DiLoreto, Aubrey Flowers, Kira Reich, Stanley Dowell, and Zachary Pope

This interview is being conducted as part of a research study by the University of Tennessee Health Science Center. This interview is designed to assess the perspectives of clinic staff on the integration and use of the lay health coaches and perceptions of their effects on patient care. This interview will take about 15 - 20 minutes to complete. Participation is voluntary and will not adversely affect your rights or your employment status. There will be no identifiable information about you shared as a part of this process. All information collected in the study will be kept confidential, and all recorded materials on a password-protected computer and in a locked file cabinet only accessible to key study personnel. Once transcribed by myself, Kira Reich, the recordings will be deleted. If you have any questions or concerns, please contact Kira Reich or Dr. Jim Bailey at 901-448-2475. Thank you for your participation.

Clinic Personnel Perspectives on the Integration of Lay Health Coaches Interview Guide:

1)      What have been your professional successes in working with patients on lifestyle management issues? The challenges?

2)      What has been your experience overall having the lay health coaches in your practice?

3)      How have the lay health coaches impacted you and your patients?

4)      What have been the barriers or challenges in referring patients to the lay health coaches? What have been the benefits?

5)      How would you compare and contrast working with a lay health coach versus a more clinically trained staff member?

6)      Do you feel that the lay health coach in your clinic has received adequate training?

7)      What do you know/think about the motivational interviewing approach that your lay health coach uses?

8)      What are your suggestions about how lay health coaches could perform more effectively or integrate into the patient care team more successfully?

What other comments do you have about lay health coaches?
